# Pseudo-scab

**Published:** 1899-03

**Authors:** S. D. Brimhall

**Affiliations:** Field Veterinarian, State Board of Health, Minnesota


### PSEUDO-SCAB.

By S. D. Brimhall, V.M.D.,
FIELD VETERINARIAN, STATE BOARD OF HEALTH, MINNESOTA.

A MILL-OWNER in the south-central part of Minnesota, who for a number of years past has fed several thousand sheep each winter, shipped 5200 sheep from the Montana ranges in the latter part of November. Soon after their arrival at the feeding-pens the owner noticed that they were badly troubled with ticks, and had them dipped in scab cure. There was nothing wrong with the wool at this time, but shortly after the dipping a great many of the sheep were noticed "taggy," especially about the neck, although there were tags on all parts of the body. On close inspection a great many spots devoid of wool were found. These spots varied greatly in size—those on the body were generally circular in outline, while on the neck they had no definite form; the smallest were about the size of a five-cent piece, and the largest the size of a man's hand.

In my search for the cause of this loss of wool I noticed that the skin in the centre of these denuded areas had either a sore covered with a scab or a scar denoting the presence of a sore at that point at some time previous. About the neck were numerous sores and furrows underneath the skin, containing pus, and in some places there were small abscesses situated in the muscles and opening through the skin. The deep sores and abscesses proved to be due to the penetration through the skin of a head of spear grass (*Stipa*

*spartea*). The more superficial sores were caused by the penetration into the skin of a beard of squirrel-tail grass (*Hordium jubatum*). The loss of wool about the face was caused by the discharge from the eyes, due to the irritating presence of a piece of this same squirrel-tail grass.

These cases I consider of especial interest because of their similarity to true scab. No one who had ever experienced the loss caused by scab would have been willing to buy any of these sheep to put with healthy ones.

